# On a New Iterative Scheme without Memory with Optimal Eighth Order

**DOI:** 10.1155/2014/727490

**Published:** 2014-08-28

**Authors:** M. Sharifi, S. Karimi Vanani, F. Khaksar Haghani, M. Arab, S. Shateyi

**Affiliations:** ^1^Department of Mathematics, Islamic Azad University, Shahrekord Branch, Shahrekord, Iran; ^2^Department of Mathematics and Applied Mathematics, University of Venda, Thohoyandou 0950, South Africa

## Abstract

The purpose of this paper is to derive and discuss a three-step iterative expression for solving nonlinear equations. In fact, we derive a derivative-free form for one of the existing optimal eighth-order methods and preserve its convergence order. Theoretical results will be upheld by numerical experiments.

## 1. Introduction

Assume that *f* : *D*⊆*R* → *R* is sufficiently smooth and that *α* ∈ *D* is its simple zero; that is, *f*(*α*) = 0. This paper concerns with numerical solution of nonlinear scalar equations by iterative expressions. Considering a known optimal eighth-order method with derivative and the conjecture of Cordero and Torregrosa [1], we construct a family of derivative-free methods without memory for solving a nonlinear equation.

To shortly review the literature, we remind readers of the following. Kung and Traub in [2] have provided a class of *n*-step derivative-involved methods including *n* evaluations of the function and one of its first derivatives per full iteration to reach the convergence rate 2^*n*^. They also have given a *n*-step derivative-free family of one parameter (consuming *n* + 1 evaluations of the function) to again achieve the optimal convergence rate 2^*n*^.

**Remark 1 (Kung-Traub's conjecture [2]).**

Multipoint iterative methods without memory, requiring *d* + 1 function evaluations per iteration, have the order of convergence at most 2^*d*^. Multipoint methods which satisfy the Kung-Traub conjecture (still unproved) are called optimal methods.

Some well-known methods with eighth order of convergence can be found at [3]. As another example, Liu and Wang [4] suggested some optimal eighth-order methods using four evaluations per full cycle (*β*_1_, *β*_2_ ∈ *R*) in what follows:

(1) yn=xn−f(xn)f′(xn),zn=yn−f(yn)f′(xn)4f(xn)−f(yn)4f(xn)−9f(yn),xn+1= zn−f(zn)f′(xn)[8f(yn)4f(xn)−11f(yn)       +(1+f(zn)3f(yn)−β1f(zn))3       +4f(zn)f(xn)+β2f(zn)],

where the efficiency index is 1.682. Reference [4] also suggested the following three-step approach (*α*_1_, *α*_2_ ∈ *R*) with the same number of evaluations and efficiency index:

(2) yn=xn−f(xn)f′(xn),zn=yn−f(yn)f′(xn)f(xn)f(xn)−2f(yn),xn+1= zn−f(zn)f′(xn)[(f(xn)−f(yn)f(xn)−2f(yn))2       +f(zn)f(yn)−α1f(zn)       +4f(zn)f(xn)+α2f(zn)].

In what follows, in Section 2, the main derivation is provided to design a new derivative-free family with optimal eighth-order convergence for nonlinear equations. Therein, we confirm the conjecture of Cordero-Torregrosa as well.  Section 3 illustrates the accuracy of the new obtained three-step family of iterative methods by comparing the results for some nonlinear test functions. Finally, in Section 4, a conclusion will be drawn.

## 2. A New Derivative-Free Family

There are a number of papers (see, e.g., [1] and the references therein) about the idea of removing derivatives from the iteration function in order to avoid defining new functions and calculate iterates only by using the function that describes the problem and also trying to preserve the convergence order. The interest of these methods is to be applied on nonlinear equations when there are many problems for obtaining and evaluating the derivatives involved or when there is no analytical function to derive.

Hence, our focus in this work is to derive a method without the use of derivatives for nonlinear equations.

**Remark 2 (Cordero and Torregrosa's conjecture [1]).**

Every time that one applies the approximation of the derivative *f*′(*x*_*n*_) ≈ *f*[*x*_*n*_, *w*_*n*_], with *w*_*n*_ = *x*_*n*_ + *βf*(*x*_*n*_)^*l*^, on an optimal method with the order 2*q*, one needs *l* ≥ *q* for preserving the order of convergence.

We begin by reminding the readers of the three-step iterative method without memory proposed in [5] with optimal eighth order of convergence:

(3) yn=xn−f(xn)f′(xn),zn= xn−(f(xn)f′(xn)f(xn)f(xn)−f(yn))×(1+(f(yn)f(xn))2+3(f(yn)f(xn))3),xn+1= zn−(f(zn)f[zn,yn]+f[zn,xn,xn](zn−yn))×(1+2f(zn)f(xn)−18(f(yn)f(xn))4+(f(zn)f(yn))3).

The main aim is to follow Remark 2 and to present a derivative-free form of (3) with optimal eighth order of convergence. Therefore, using the approximation *w*_*n*_ = *x*_*n*_ + *βf*(*x*_*n*_)^3^, we present the following formulation (*β* ∈ *R*∖{0}):

(4) yn=xn−f(xn)f[xn,wn],  wn=xn+βf(xn)3,zn= xn−(f(xn)f[xn,wn]f(xn)f(xn)−f(yn))×(1+(f(yn)f(xn))2+3(f(yn)f(xn))3),xn+1= zn−(f(zn)f[zn,yn]+φzn,xn,xn(zn−yn))×(1+2f(zn)f(xn)−18(f(yn)f(xn))4+(f(zn)f(yn))3),

wherein

(5) $$\begin{array}{c} \varphi_{z_{n},x_{n},x_{n}}=\frac{f\left[z_{n},x_{n}\right]-f\left[x_{n},w_{n}\right]}{z_{n}-x_{n}}. \end{array}$$

We shall see that the order of convergence for (4) reaches to the optimal case, that is, 8, with only four function evaluations per full iteration, which means that the proposed uniparametric family of derivative-free methods possesses the high efficiency index 1.682 and can be viewed as the derivative-free formulation of (3).

**Theorem 3 .**

Let *α* ∈ *D* be a simple zero of a sufficiently differentiable function *f* : *D* ⊂ *R* → *R* for an open interval *D*, which includes *x*_0_ as an initial approximation of *α*. Then, the family of derivative-free methods (4) is of optimal order eight.

**Proof**

To find the asymptotic error constant of (4) where *c*_*j*_ = *f* ^(*j*)^(*α*)/*j*!, *j* ≥ 1, we expand any terms of (4) around the simple root *α* in the *n*th iterate. Thus, we write

(6) f(xn)= c1en+c2en2+c3en3+c4en4+c5en5+c6en6+c7en7+c8en8+O(en9),

where *e*_*n*_ = *x*_*n*_ − *α* and

(7) f(wn)= c1bn+c2bn2+c3bn3+c4bn4+c5bn5+c6bn6+c7bn7+c8bn8+O(bn9),

wherein *b*_*n*_ = *w*_*n*_ − *α*. Hence, we obtain

(8) xn−f(xn)f[xn,wn]−α  =c2en2c1+2(−c22+c1c3)en3c12+⋯+O(en9).

In the same vein, we have

(9) $$\begin{array}{c} f\left(y_{n}\right)=c_{2}e_{n}^{2}+\left(-\frac{2c_{2}^{2}}{c_{1}}+2c_{3}\right)e_{n}^{3}+\cdots +O\left(e_{n}^{9}\right), \end{array}$$

and for the second substep, we have

(10) zn−α =−c2c3c12en4  +(−βc15c22+9c24+2c1c22c3−2c12(c32+c2c4))c14en5  +⋯+O(en9).

At this time, Taylor's series expansion of *f*(*z*_*n*_) around the root is needed. We find that

(11) f(zn) =−c2c3en4c1  +(−βc15c22+9c24+2c1c22c3−2c12(c32+c2c4))c13en5  +⋯+O(en9),

and subsequently

(12) φzn,xn,xn= c2+2c3en+(βc13c2+3c4)en2+3βc12(c22+c1c3)en3+⋯+O(en9).

Considering these Taylor's series expansions in the last step of (4) will result in the following final error equation:

(13) $$\begin{array}{c} e_{n+1}=-\frac{c_{2}^{2}c_{3}\left(c_{2}\left(\beta c_{1}^{4}-2c_{3}\right)+c_{1}c_{4}\right)}{c_{1}^{5}}e_{n}^{8}+O\left(e_{n}^{9}\right). \end{array}$$

This shows that the iterative family of derivative-free methods without memory (4) is of optimal order eight. The proof is complete.

**Remark 4 .**

Theorem 3 clearly supports the conjecture of Cordero-Torregrosa for providing low-complexity derivative-free iterative methods without memory out of optimal methods with derivative.

Note that each method of (4) reaches the efficiency index $\sqrt[{4}]{8}\approx 1.682$, which is greater than $\sqrt[{3}]{4}\approx 1.587$ of optimal fourth-order techniques and $\sqrt[{2}]{2}\approx 1.424$ of optimal Newton's method. It has also the same computational efficiency index with (1), (2), and (3).

**Remark 5 .**

It must be remarked that, firstly, the paper [6] studied the multipoint iterative schemes using divided differences for self-acceleration of classical methods.

We here state that the free nonzero parameter *β* in (4) gives us the ability to increase the convergence *R*-order of (4) more. Such an acceleration in *R*-order is known as with memorization (see, e.g., [7]) according the classification of Traub [8] for nonlinear solvers. To be more precise, choosing

(14) $$\begin{array}{c} \beta =\frac{2c_{2}c_{3}-c_{1}c_{4}}{c_{1}^{4}c_{2}} \end{array}$$

would yield an acceleration of convergence.

Anyhow, since the simple zero *α* and subsequently *c*_*j*_ are not known, one should give an approximation for (14) using an approximation polynomial *A*(*t*) ≈ *f*(*t*) in the domain *D*. Toward this goal, if we consider *A*(*t*) to be Newton's interpolatory polynomial of fourth degree passing through the five available nodes *x*_*n*−1_, *w*_*n*−1_, *y*_*n*−1_, *z*_*n*−1_, and *x*_*n*_ at the end of each cycle, then one has the following approximation:

(15) $$\begin{array}{c} \beta_{n}=\frac{4A^{\left(3\right)}\left(x_{n}\right)-A^{\prime }\left(x_{n}\right)A^{\left(4\right)}\left(x_{n}\right)/A^{\prime \prime }\left(x_{n}\right)}{12A^{\prime }{\left(x_{n}\right)}^{4}}, \end{array}$$

using a suitable *β*_0_. Consequently, one is able to derive the following accelerated iterative method with memory:

(16) βn=4A(3)(xn)−A′(xn)A(4)(xn)/A′′(xn)12A′(xn)4,yn=xn−f(xn)f[xn,wn],  wn=xn+βnf(xn)3,zn= xn−(f(xn)f[xn,wn]f(xn)f(xn)−f(yn))×(1+(f(yn)f(xn))2+3(f(yn)f(xn))3),xn+1= zn−(f(zn)f[zn,yn]+φzn,xn,xn(zn−yn))×(1+2f(zn)f(xn)−18(f(yn)f(xn))4+(f(zn)f(yn))3).

Obviously, if fewer nodes are used for the interpolating polynomial, slower acceleration is achieved. An increase of convergence is achieved in this way without additional functional evaluations, making the proposed root solvers (16) efficient. This acceleration will be seen in Section 3.

**Theorem 6 .**

Let the function *f*(*x*) be sufficiently differentiable in a neighborhood of its simple zero *α*. If an initial approximation *x*_0_ is sufficiently close to *α*, then the *R*-order of convergence of (16) is at least $4+\sqrt{17}$.

**Proof**

Let {*x*_*n*_} be a sequence of approximations generated by an iterative method with order *p*. The error relation with the self-accelerating parameter *β* = *β*_*n*_ for (16) is in what follows:

(17) $$\begin{array}{c} e_{n+1}=x_{n+1}-\alpha \sim c_{n,8}e_{n}^{8}, \end{array}$$

wherein *c*_*n*,8_ is the asymptotic error constant. Using a symbolic computation and (13), we attain that

(18) $$\begin{array}{c} \frac{2c_{2}c_{3}-c_{1}c_{4}}{c_{1}^{4}c_{2}}\sim e_{n-1}. \end{array}$$

Substituting the value of (2*c*_2_ *c*_3_ − *c*_1_ *c*_4_)/(*c*_1_^4^ *c*_2_) from (18) in (17), one may obtain

(19) $$\begin{array}{c} e_{n+1}\sim e_{n-1}e_{n}^{8}. \end{array}$$

Thus, it is easy to obtain

(20) $$\begin{array}{c} e_{n}^{p}\sim A^{-1/p}Ce_{n}^{8+1/p}, \end{array}$$

wherein *A* and *C* are two constants and subsequently

(21) $$\begin{array}{c} p=8+\frac{1}{p}, \end{array}$$

with two solutions $\left\{4-\sqrt{17},4+\sqrt{17}\right\}$. Clearly the value for $p=4+\sqrt{17}\approx 8.12311$ is acceptable and would be the convergence *R*-order of the method (16) with memory. The proof is complete.

## 3. Numerical Testing

The objective of this section is to provide a comparison between the presented schemes and the already known methods in the literature.

For numerical reports here, we have used the optimal eighth-order three-step method (1) as (LW8) with *β*_1_ = *β*_2_ = 0, the optimal eighth-order three-step method (3) as (SM8), our optimal three-step eighth-order method (4) with *β* = −0.0001, and the accelerated method with memory (16) denoted by (APM) with *β*_0_ = −0.0001.

The results are summarized in Tables 1 and 2 after some full iterations. As they show, novel schemes are comparable with all of the methods. All numerical instances were performed by Mathematica 8 using 1000 fixed floating point arithmetic [9].

We have computed the root of each test function for the initial guess *x*_0_ while the iterative schemes were stopped when |*f*(*x*_*n*_)|≤10^−150^. As can be seen, the obtained results in Tables 1 and 2 are in harmony with the analytical procedure given in Section 2.

The computational order of convergence (COC) has also been computed by

(22) $$\begin{array}{c} \text{COC}=\frac{\ln\left|f\left(x_{n}\right)/f\left(x_{n-1}\right)\right|}{\ln\left|f\left(x_{n-1}\right)/f\left(x_{n-2}\right)\right|}. \end{array}$$

**Example 7 .**

In this test, we compare the behavior of different methods for finding the complex solution of the following nonlinear equation:

(23) $$\begin{array}{c} f\left(x\right)=\left(-1+2I\right)+\frac{1}{x}+x+\sin\left(x\right), \end{array}$$

using the initial approximation *x*_0_ = 1 − 3*I* where *α* = 0.28860 ⋯ −1.24220 ⋯ *I*. The results for this test are given in Table 1.

**Example 8 .**

We here compare the behavior of different methods for finding the solution of

(24) $$\begin{array}{c} g\left(x\right)=\left(-2+x\right)\sin\left(\tanh\left(x\right)\right), \end{array}$$

using the initial approximation *x*_0_ = 1.0 where *α* = 2. The results for this test are given in Table 2.

It should be mentioned that our method (4) cannot be easily extended for nonlinear systems. The reason is that the weight functions used in (4) do not contain a finite difference operator in the denominators. Such an extension might be pursued for future studies. However, a simple extended version of (4) for the *N*-dimensional case can be written in what follows:

(25) $$\begin{array}{c} y^{\left(n\right)}=x^{\left(n\right)}-J_{x,w}^{-1}F\left(x^{\left(n\right)}\right),\;n=0,1,2,\ldots ,\\ z^{\left(n\right)}=y^{\left(n\right)}-\left[J_{x,y}^{-1}J_{x,w}-I\right]\left[J_{x,w}^{-1}F\left(x^{\left(n\right)}\right)\right],\\ x^{\left(n+1\right)}=z^{\left(n\right)}-J_{y,z}^{-1}F\left(z^{\left(n\right)}\right), \end{array}$$

wherein **w** ^(*n*)^ = **x** ^(*n*)^ + *F*(**x** ^(*n*)^) and it possesses only fifth order of convergence. Note that the extended version of Steffensen's method has been written by

(26) $$\begin{array}{c} x^{\left(n+1\right)}=x^{\left(n\right)}-J_{x,w}^{-1}F\left(x^{\left(n\right)}\right),\;n=0,1,2,\ldots , \end{array}$$

wherein

(27) Jx,w=J(x(n),βH(n))=(F(x(n)+H(n)e1)−F(x(n)),…,  F(x(n)+H(n)eN)−F(x(n)))H(n)−1,

with *H* ^(*n*)^ = diag⁡(*βf*_1_(**x** ^(*n*)^),…, *βf*_*N*_(**x** ^(*n*)^)). Now we apply (25) to solve a nonlinear integral equation, and keeping the rate of convergence at eight will remain as an open problem for future works.

**Example 9 .**

Consider the mixed Hammerstein integral equation [10]:

(28) $$\begin{array}{c} x\left(s\right)=1+\frac{1}{5}\overset{1}{\underset{0}{\int }}G\left(s,t\right)x{\left(t\right)}^{3}dt, \end{array}$$

where *x* ∈ *C*[0,1], *s*, *t* ∈ [0,1], and the kernel *G* is given by

(29) $$\begin{array}{c} G\left(s,t\right)=\{\begin{array}{cc} \left(1-s\right)t, & t\leq s,\\ s\left(1-t\right), & t>s. \end{array} \end{array}$$

In order to solve this nonlinear integral equation, we transform the above equation into a finite-dimensional problem by using Gauss-Legendre quadrature formula given as

(30) $$\begin{array}{c} \overset{1}{\underset{0}{\int }}f\left(t\right)dt\approx \overset{t}{\underset{j=1}{\sum }}w_{j}f\left(t_{j}\right), \end{array}$$

where the abscissas *t*_*j*_ and the weights *w*_*j*_ are determined for *t* = 10 by Gauss-Legendre quadrature formula. Denoting the approximation of *x*(*t*_*i*_) by *x*_*i*_  (*i* = 1,2, ⋯, *t*), we obtain the system of nonlinear equations

(31) $$\begin{array}{c} f\left(x_{1},\ldots ,x_{t}\right)=5x_{i}-5-\overset{t}{\underset{j=1}{\sum }}a_{ij}x_{j}^{3}=0, \end{array}$$

where, for *i* = 1,2,…, *t*, we have

(32) $$\begin{array}{c} a_{ij}=\{\begin{array}{cc} w_{j}t_{j}\left(1-t_{i}\right), & \text{if}\;\;j\leq i,\\ w_{j}t_{i}\left(1-t_{j}\right), & \text{if}\;\;i<j, \end{array} \end{array}$$

wherein the abscissas *t*_*j*_ and the weights *w*_*j*_ are known.

Using the initial approximation **x** ^(0)^ = (0.5,…, 0.5)^*T*^, we apply the proposed method (25) denoted by PMS with *β* = 0.001 which is multiplication-rich to find the final solution vector of the nonlinear integral equation (31).  Table 3 shows the residuals in *l*_2_ norm, when *t* = 10 is the size of the nonlinear system of equations.

## 4. Concluding Remarks

Solving nonlinear equations is a classical problem which has interesting applications in various branches of science and engineering (see, e.g., [11]). In this study, we have described an iterative method without memory to find a simple root *α* of a nonlinear equation *f*(*x*) = 0 on an open interval *D*.

The derived scheme was developed by applying the conjecture of Cordero-Torregrosa and it was proved that it converges to the simple zero of a nonlinear equation with optimal eighth order of convergence. This shows that it has the optimal efficiency index 1.682. We, furthermore, discussed how to increase the *R*-order of convergence via with memorization. Some examples have also been included to support the theoretical parts.

## Figures and Tables

**Table 1:** Results of comparisons for Example 7.

Methods	|*f*(*x*_1_)|	|*f*(*x*_2_)|	|*f*(*x*_3_)|	|*f*(*x*_4_)|	COC
LW8	0.85460	4.8410 × 10^−7^	2.8515 × 10^−57^	4.1317 × 10^−459^	8.00000
SM8	0.85215	1.4818 × 10^−8^	3.3414 × 10^−70^	2.2345 × 10^−563^	8.00000
PM	0.64289	2.2594 × 10^−9^	9.7768 × 10^−77^	1.2017 × 10^−615^	8.00000
APM	0.64289	1.2590 × 10^−9^	2.0944 × 10^−79^	2.9252 × 10^−646^	8.12358

**Table 2:** Results of comparisons for Example 8.

Methods	|*f*(*x*_1_)|	|*f*(*x*_2_)|	|*f*(*x*_3_)|	|*f*(*x*_4_)|	COC
LW8	0.080790	4.3442 × 10^−14^	6.3406 × 10^−112^	1.3057 × 10^−894^	8.00000
SM8	2.0294	2.2403 × 10^−9^	1.1696 × 10^−74^	6.4533 × 10^−597^	8.00000
PM	2.0357	2.2049 × 10^−9^	1.0295 × 10^−74^	2.3266 × 10^−597^	8.00000
APM	2.0357	9.1086 × 10^−10^	7.2327 × 10^−78^	1.3852 × 10^−631^	8.13093

**Table 3:** Results of comparisons for Example 9.

Methods	||*f*(*x*_1_)||	||*f*(*x*_2_)||	||*f*(*x*_3_)||	||*f*(*x*_4_)||	COC
PMS	23.6907	2.05639 × 10^−8^	6.73602 × 10^−48^	2.554 × 10^−245^	4.99994
